# VARIFI—Web-Based Automatic Variant Identification, Filtering and Annotation of Amplicon Sequencing Data

**DOI:** 10.3390/jpm9010010

**Published:** 2019-02-01

**Authors:** Milica Krunic, Peter Venhuizen, Leonhard Müllauer, Bettina Kaserer, Arndt von Haeseler

**Affiliations:** 1 Center for Integrative Bioinformatics Vienna, Max F. Perutz Laboratories, University of Vienna, Medical University of Vienna, Dr. Bohrgasse 9, 1030 Vienna, Austria; arndt.von.haeseler@unvie.ac.at; 2 Department of Applied Genetics und Cell Biology, University of Natural Resources and Life Sciences, Muthgasse 18, 1190 Vienna, Austria; peter.venhuizen@boku.ac.at; 3 Institute of Pathology, Medical University Vienna, Währinger Gürtel 18-20, 1090 Vienna, Austria; leonhard.muellauer@meduniwien.ac.at (L.M.); bettina.kaserer@meduniwien.ac.at (B.K.); 4 Bioinformatics and Computational Biology, Faculty of Computer Science, University of Vienna, 1090 Vienna, Austria

**Keywords:** personalized medicine, cancer, amplicon sequencing, variant finding, pipeline

## Abstract

Fast and affordable benchtop sequencers are becoming more important in improving personalized medical treatment. Still, distinguishing genetic variants between healthy and diseased individuals from sequencing errors remains a challenge. Here we present VARIFI, a pipeline for finding reliable genetic variants (single nucleotide polymorphisms (SNPs) and insertions and deletions (indels)). We optimized parameters in VARIFI by analyzing more than 170 amplicon-sequenced cancer samples produced on the Personal Genome Machine (PGM). In contrast to existing pipelines, VARIFI combines different analysis methods and, based on their concordance, assigns a confidence score to each identified variant. Furthermore, VARIFI applies variant filters for biases associated with the sequencing technologies (e.g., incorrectly identified homopolymer-associated indels with Ion Torrent). VARIFI automatically extracts variant information from publicly available databases and incorporates methods for variant effect prediction. VARIFI requires little computational experience and no in-house compute power since the analyses are conducted on our server. VARIFI is a web-based tool available at varifi.cibiv.univie.ac.at.

## 1. Introduction

Individualized medical genetics assists in therapy decisions in a way that it helps practitioners to prescribe an appropriate therapy to the particular patient. Benchtop next-generation sequencing (NGS) machines (e.g., MiniSeq and MiSeq from Illumina, Personal Genome Machine (PGM) using Ion Torrent technology from Thermo Fisher Scientific) facilitate patient genetic characterization in a routine clinical setting. By discovering disease-related genes, targeted NGS has become an important sequencing method in clinical diagnostics [1,2,3,4]. Amplicon sequencing is one type of targeted sequencing where, before sequencing, PCR amplification of relevant parts of targeted genomic regions occurs. Targeted sequencing lowers sequencing costs, runtime and the time required for downstream analysis of the sequencing output. It produces enough coverage for the genomic regions of interest to detect single nucleotide polymorphisms (SNPs) and other variants. Thus, amplicon sequencing is well suited as a routine clinical genetic diagnostic tool.

The next step is the downstream analysis of the amplicon sequencing data. This step involves finding differences between a sample of interest (usually in a disease state) and a control sample (in a reference, healthy state). Apart from distinguishing genetic variants from errors, the next critical step in the downstream analysis is to inspect medical relevance of found variants by utilizing variant annotation and impact prediction tools. Once the medical relevance of a given variant is determined, one can move on to search for available drugs which target the genetic variants. However, downstream analysis requires computational skills and enough computational resources to do this efficiently. In a routine clinical diagnostic procedure, the downstream analysis needs to be fast and reproducible.

To fulfill the mentioned requirements, we developed VARIFI, a freely available pipeline for the automatic variant identification, filtering and annotation of data produced by amplicon sequencing.

We followed suggestions from recent publications [5,6] and used a combination of aligners and mutation (variant) callers to gain higher accuracy. VARIFI requires minimal computational experience and no in-house computing power since the analyses are conducted on our server. Running VARIFI ensures that the sequenced data are all processed in a coherent and standardized way, which in turn facilitates reproducibility and comparability between different samples. To successfully distinguish genetic variants between healthy and diseased individuals from sequencing errors, the relevant parameters in VARIFI were optimized from the analysis of more than 170 amplicon-sequenced cancer samples produced on the PGM and then verified by Sanger sequencing. The required input from the user is greatly simplified and contains only two input files: sequencing data (reads) in bam or fastq format and a list of sequenced targeted regions (amplicons) in bed file format.

**Figure 1:**
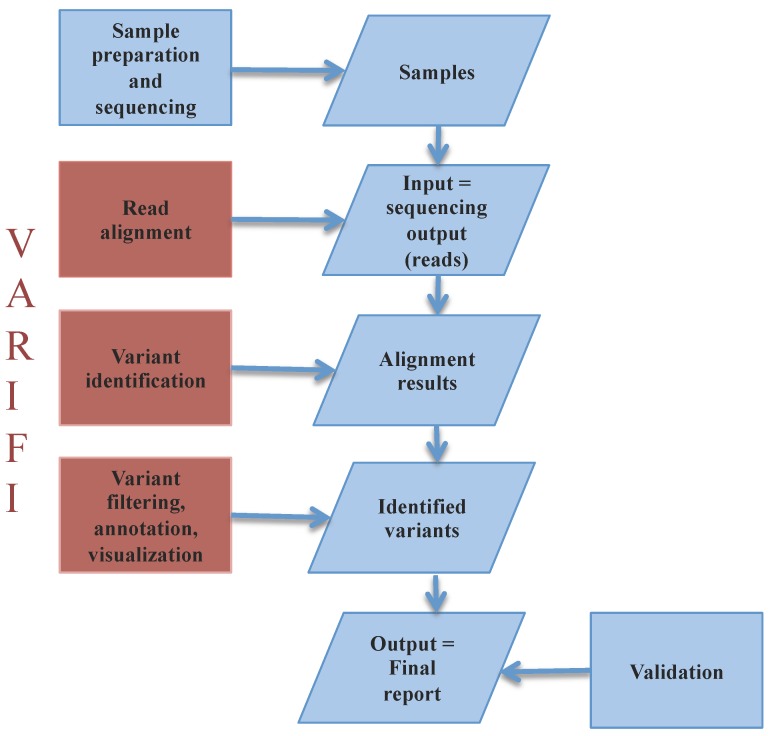
Complete variant analysis workflow from the sample preparation to the variant validation. Processes are presented using rectangles: VARIFI processes are in red, and laboratory experiments which we used to develop and optimize VARIFI are in blue rectangles. Parallelograms show input/output data of these processes. Arrows show the process and data generation flow. After sample preparation and sequencing, VARIFI starts by aligning the reads against the human genome reference and continues with variant identification in the alignment results. Detected variants are then filtered, annotated and prepared for visualization. VARIFI output is a list of filtered and annotated variants displayed in a final report. We used variant validation to optimize and evaluate VARIFI processes.

VARIFI (Figure 1) first maps the reads on the human reference genome (hg19) and continues with variant identification. VARIFI outputs a list of annotated variants: SNPs and insertions and deletions (called indels). Each variant can be visually inspected. VARIFI assigns a confidence score to every identified variant, which can be used as a prioritizing criterion in selecting the most reliable variants. In addition, VARIFI applies variant filters for biases associated with the sequencing technologies (e.g., incorrectly identified homopolymer-associated indels with Ion Torrent). VARIFI automatically extracts variant information from publicly available databases and incorporates methods for variant effect prediction. To the best of our knowledge, there is no available non-commercial variant identification pipeline that combines all of the abovementioned features. We compared the results of VARIFI with the widely used commercial tool Ion Reporter from Thermo Fisher Scientific, and it was found that VARIFI has greater specificity and sensitivity.

## 2. Materials and Methods

### 2.1. Sample Preparation and Amplicon Sequencing

DNA was extracted from paraffin-embedded tissue blocks with a QIAamp Tissue Kit^TM^ (Qiagen, Hilden, Germany). A total of 10 ng DNA per sample was utilized for sequencing. The DNA library was generated by multiplex polymerase chain reaction (PCR) with two panels—Ion AmpliSeq Cancer Hotspot Panel v2^TM^ and ColonLung Panel v2^TM^ (both from Thermo Fisher Scientific, Waltham, MA, USA). The first panel covers mutation hotspots of 50 genes, mostly oncogenes and tumor suppressor genes, which are frequently mutated in tumors, whereas the ColonLung Panel covers the mutational hotspots of 22 genes, which are altered in colon and lung cancers. Template preparation was carried out by emulsion PCR with Ion One Touch^TM^ or Ion Chef^TM^ instruments (Thermo Fisher Scientific). Sequencing was performed with an Ion Torrent PGM^TM^ (Thermo Fisher Scientific).

### 2.2. Bioinformatic Analysis

#### 2.2.1. Read Alignment

We mapped the reads against the human genome reference (hg19) using three aligners: Burrows-Wheeler Aligner (bwa, v0.6.1-r104) [7], bowtie2 (v2.2.5) [8] and NextGenMap (v0.4.10-pre) [9]. We used them with default parameters. NextGenMap was used with several additional options: identity (-i) was set to 0.85, the maximum number of consecutive indels allowed (-C) was set to 120 and we used alignment algorithms that support affine gap costs (--affine).

#### 2.2.2. Alignment Post-Processing

We replaced read groups in aligned reads (BAM files) using Picard tools (http://broadinstitute.github.io/picard) option AddorReplaceReadGroups. The aligned reads were then indexed using SAMtools (v1.1) [10]. Local realignment around insertions and deletions and quality base score recalibration were performed using the Genome Analysis Tool Kit (GATK, v2.6) [11].

#### 2.2.3. Variant Identification

To call variants (SNPs and indels) in aligned reads files, we used two state-of-the-art variant callers: UnifiedGenotyper from GATK and SAMtools together with its bcftools. The options used for UnifiedGenotyper were: -dcov set to 2000, --standard_min_confidence_threshold_for_calling set to 30.0, --standard_min_confidence_threshold_for_emitting set to 10, -glm set to BOTH and for option --dbsnp. We used human_9606 variants from the dbSNP database [12].

SAMtools mpileup (v0.1.18) and vcfutils.pl from SAMtools bcftools (v0.1.18) were used as the second variant caller. The option we set for SAMtools mpileup was a minimum-mapping quality (-q) of 20.

#### 2.2.4. Variant Filtration

The variants called using GATK were first separated into SNPs and indels using SelectVariant. SNPs to be filtered out were labeled using VariantFiltration with the following filter expressions: --clusterWindowSize = 10, “MQ0 ≥ 4 && ((MQ0/(1.0 * DP)) > 0.1)”, “DP < 5”, “QUAL < 50.0”, “QD < 0.7”, “FS > 60.0” and --missingValuesInExpressionsShouldEvaluateAsFailing, where the full parameters’ names are: MQ0: MappingQualityZero, DP: DepthPerSampleHC, QUAL: Quality, QD: QualByDepth, FS: FisherStrand. Only for bwa, the FS setting was: “FS > 590.0”. Correction was made based on Sanger results. Indels to be filtered out were labeled using VariantFiltration with the filter expressions: “QD < 2.5 || ReadPosRankSum < -20.0 || FS > 200.0”, “--missingValuesInExpressionsShouldEvaluateAsFailing”. All variants (labeled for filtering and unlabeled) were then combined by the GATK CombineVariants tool. Labeled SNPs and indels were filtered out using our scripts. We retained variants if at least one aligner-GATK combination did not label the variant.

The variants called using SAMtools were filtered using the following options for vcfutils.pl: minimum coverage 2, maximum coverage 4000, SNP within 0 bp around a gap to be filtered (-w = 0), window size for filtering adjacent gaps, -W = 0.

The in-house developed filter for incorrectly identified homopolymer-associated indels is explained in the Results Section.

#### 2.2.5. Merging Variants

Variants called by GATK and SAMtools were combined using vcf-merge and our in-house developed tools.

#### 2.2.6. Variant Annotation

Merged variants were annotated with ANNOVAR [13] (v11Feb2013, databases: dbSNP [12], 1000 genome [14]; variant effect predictors: PolyPhen-2 [15], MutationTaster [16], PhyloP [17], GERP++ [18], SIFT [19]) and the COSMIC database (v63) [20].

#### 2.2.7. Visualization

For variant visualization we used Integrative Genomics Viewer (IGV, v2.3.5) [21].

#### 2.2.8. Amplicon Coverage and Plotting

To calculate the number of reads covering amplicons, we used our Java, python and R scripts. Plots were made using R (v3.3.3).

### 2.3. Variant Validation—Sanger Sequencing

Nonsynonymous mutations detected with the Ion Torrent PGM^TM^ were verified by capillary sequencing. PCR primers flanking the DNA mutation were designed. DNA was amplified by PCR with Jump Start^TM^ REDtaq^R^ Ready Mix^TM^ (Sigma-Aldrich, Vienna, Austria). PCR products were cleaned with ExoSAP-IT (Affymetrix, Santa Clara, CA, USA). The sequencing of PCR products was carried out using a BigDye^R^ Terminator v1.1 Cycle Sequencing Kit (Applied Biosystems, Foster City, CA, USA). The resulting DNA fragments were purified using a DyeEx 96 kit (Qiagen) and sequenced using a 3500 Genetic Analyzer (Applied Biosystems). Sequence analysis employed the SeqScape Version 2.7 software (Applied Biosystems).

### 2.4. VAIRFI Performance Calculations and Comparison with Ion Reporter

In order to calculate sensitivity and specificity, first we determined true positive (TP), true negative (TN), false positive (FP) and false negative (FN) variants. To do that, we used the Sanger method to sequence several exons of the *TP53* gene in 13 samples analyzed with Ion AmpliSeq Cancer Hotspot Panel v2^TM^ and six samples using ColonLung Panel v2^TM^ (Life Technologies, Carlsbad, CA, USA). In parallel, we searched for the variants in the same exons with both pipelines—VARIFI and Ion Reporter (version 5.2). The information on how many and which exons were sequenced in each sample are shown in Table S1. We calculated the sensitivity and specificity for each panel and for each pipeline, i.e., VARIFI and Ion Reporter, separately. If the variant was found by a pipeline and confirmed by the Sanger method, that variant was considered a TP. TNs were defined as positions where neither Sanger sequencing nor a pipeline found a variant. If a pipeline found a variant and the Sanger method did not confirm it, then the variant was considered an FP. If Sanger sequencing detected a variant and a pipeline did not, then the variant was considered an FN. We calculated sensitivity as TP/(TP + FN), and the specificity as TN/(TN + FP).

### 2.5. Ethics Committee Approval

The study was conducted in accordance with the Declaration of Helsinki and was approved by the institutional ethic committee of the Medical University of Vienna (Nr.1541/2012).

## 3. Results

### 3.1. VARIFI Pipeline

#### 3.1.1. Input Files

VARIFI is available as a web-based tool (varifi.cibiv.univie.ac.at) and requires the user to upload two compulsory files: the sequenced reads (fastq or bam files) and a file with the list of sequenced amplicons and their positions (bed file). The user should also submit their email address, which we use to send notifications about job progress and links where the results can be downloaded.

#### 3.1.2. Processing Files

After the input files have been submitted, VARIFI maps the reads to the human genome reference (hg19). This is achieved using three state-of-the-art aligners: bwa [7], bowtie2 [8] and NextGenMap [9]. The variants are identified in the aligned files by two variant callers: SAMtools [10] and GATK [11]. Using three aligners and two variant callers, VARIFI can identify each variant by one to six combinations of the employed tools.

#### 3.1.3. Variant Filtering

VARIFI removes potential false positive variants in three filtering steps. First it removes variants identified outside of the genomic regions specified by the input bed file. In the second step, VARIFI removes variants which did not pass the general quality filters provided by the employed variant callers, e.g., filtering out variants with insufficient base quality or insufficient mapping quality (see Section 2.2.4. for filtering details). We developed a third filtering step to specifically remove falsely identified indels. This step is especially useful for data produced by some sequencing technologies, e.g., Ion Torrent, because this technology can introduce false indels in homopolymer regions [22,23]. To develop a filter that removes falsely identified indels, we introduced two filtering criteria, both based on the distribution of the deletion and insertion length at the sites of potential indels. Following Yeo et al. [24], we introduced the first criterion which we named *var*. It represents the variance of the deletion and insertion length (in bp), calculated from each read covering the potential indel site and showing a deletion or an insertion with respect to the reference genome. To control for the influence of reads, having an outlier length of the deletion or insertion, on the *var* calculation, we introduced the second criterion—*frmode*—which is the frequency of the mode of the deletion or insertion length distribution at the potential indel site. We calculated *frmode* as the percentage of reads containing the most common length of the deletion or insertion in all the reads covering the potential indel site and showing a deletion or an insertion. Potential indel sites represent genomic positions of indels which passed the first two filtering steps, indicating that they are in the genomic regions specified by an input bed file and that they have passed quality filters. An example of how *var* and *frmode* are calculated can be seen in Supplementary File S1.

**Figure 2:**
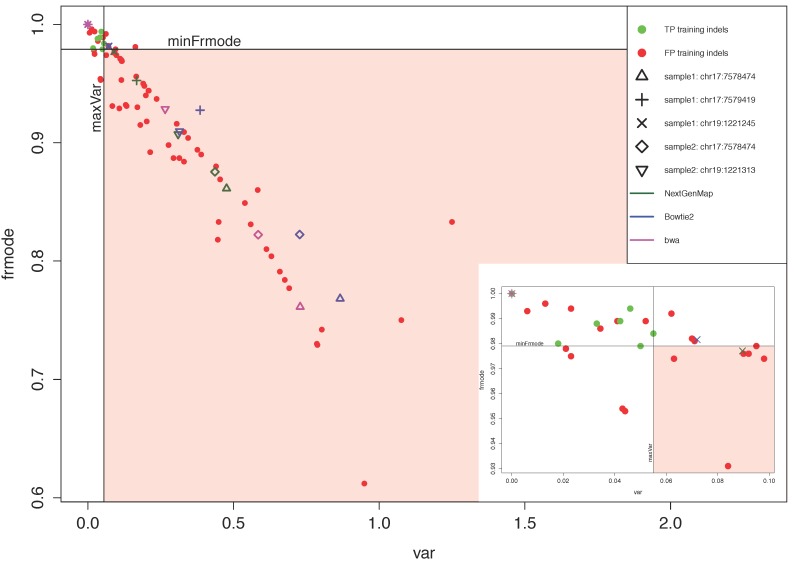
Distribution of the variance of the gap/insert length (*var*) and frequency of the most common gap/insert length (*frmode*) at the potential indel site. Green circles represent values for the parameters *var* and *frmode* for 18 true positive (TP) training indels, and red circles represent *var* and *frmode* for 90 false positive (FP) training indels. We used training indels to define threshold values for filtering parameters *var* and *frmode*. *maxVar* is the maximum value among all *var* values for training TPs. *minFrmode* is the minimum *frmode* among all *frmode* values for training TPs. We filtered out a potential indel x if *var*(x) > *maxVar* and *frmode*(x) < *minFrmode* (red shaded area). In this way, we detected all training TPs and filtered out 60% of the training FPs. To test the parameter filtering settings, we used five test indels coming from two samples (S_1_ and S_2_) which Sanger sequencing showed to be FPs. Parameters for each test indel are presented by a combination of a symbol (indel genomic position) and a color (aligner), e.g., the “+” magenta symbol represents *var* and *frmode* for an indel at the position chr17:7579419, for which the parameters were calculated from the bwa aligned file. Since the parameters were calculated for at least one aligner are in the area from which the indels were filtered out, we filtered out all five FP test indels. A small plot in the right bottom corner of the figure is an enlargement of the left upper corner of the main figure.

To calculate the threshold values for *var* and *frmode*, we used 108 training indels (indel calls) found in homopolymer regions (Figure 2) from Yeo et al. [25] as training indels. The training indels were identified in bwa-mapped files by GATK and SAMtools and then inspected by Sanger sequencing [25]. Out of 108 inspected training indels, Sanger sequencing confirmed 18 indels and we called them true positive (TP) training indels. Ninety indels we called false positive (FP) training indels, since Sanger sequencing could not confirm them [25]. For each training indel, we calculated *var* and *frmode* (Figure 2). Based on the distribution of *var* and *frmode* values for TP training indels, we selected the threshold values: *maxVar* = 0.055, which is the maximum *var* among all the values of *var* calculated for TPs, and *minFrmode* = 0.9790, which is the minimum value of *frmode* among all *frmode* values calculated for TPs. Based on these threshold values, VARIFI filters out a potential indel x if: *var*(x) > *maxVar* and *frmode*(x) < *minFrmode*. These threshold settings allowed us to detect all 18 TP training indels and to filter out 54 out of 90 (60%) FP training indels. Since VARIFI uses three aligners, for each of the potential indel sites, there are three values for *var* and three values for *frmode*. This is because *var* and *frmode* are calculated per aligned file. We used a more conservative approach by filtering out a potential indel if parameters calculated from at least one aligned file violated the thresholds of *maxVar* and *minFrmode*.

To inspect the effectiveness of filtering *maxVar* and *minFrmode* criteria on our own data, we used five potential indels from two randomly selected patient samples (S_1_ and S_2_) sequenced using PGM. S_1_ had three indel sites and S_2_ had two such sites (Tables S2 and S3, Figure 2). VARIFI initially identified those five potential indels in homopolymer regions of the two samples before proceeding to the third filtering step (with parameters *maxVar* and *minFrmode*). In S_1_, the three candidates were deletions of G, two were found in the *TP53* gene at the genomic positions chr17:7578474 and chr17:7579419, and one indel site was found in the *STK11* gene at the genomic position chr19:1221245 (Table S2). From S_2_, one potential indel site was a deletion of G in the *TP53* gene at the genomic position chr17:7578474 and the second candidate was a deletion of C in *STK11* gene at the genomic position chr19:1221313 (Table S3). Figure 2 shows three pairs of the parameters *var* and *frmode* for each potential indel site, as a combination of a symbol and a color denoting a respective aligner. A potential indel at the position chr19:1221245 (Figure 2, “x” symbol) shows why we used the more conservative approach described above—only the parameters (*var* and *frmode*) calculated using the NextGenMap aligner (green color) violated the thresholds *maxVar* and *minFrmode* and therefore showed that the indel should be filtered out. Applying *maxVar* and *minFrmode*, VARIFI filtered out all five potential indels from S_1_ and S_2_. Then we checked those indels with Sanger sequencing, which showed that all of them were initially falsely identified and should be filtered out.

#### 3.1.4. Confidence Score

As mentioned above, VARIFI can identify each variant by one to six combinations of the employed tools. The number of aligner-variant caller combinations that identify each variant was defined as the confidence score. The higher the score, the greater the confidence in the found variant. However, this does not mean that the variants found only by one combination of aligner-variant caller can be eliminated, since we show in the Table S4 that the Sanger sequencing method confirmed variants that had a confidence score of 1. In our datasets, we did not find a variant with a confidence score of 6 to be a false positive (Table S4). Thus, we recommend using a confidence score as a prioritization, but not as an elimination criterion.

#### 3.1.5. Variant Annotation

VARIFI produces a final report which presents a list of detected variants with their genomic positions and annotation details, including gene, transcript, exon and amino acid change information, reference and alternative allele information and coverage, confidence score, information about the variant from publicly available databases (dbSNP [12], 1000 genome [14], COSMIC [20]) and variant effect predictions (PolyPhen-2 [15], MutationTaster [16], PhyloP [17], GERP++ [18], SIFT [19]).

#### 3.1.6. Output Files

When VARIFI completes the analysis, the user receives an email notification with the download link information. VARIFI outputs include the following: amplicon coverage information, plots, files for visualization with IGV [21] and the final report file. The amplicon coverage information file shows the coverage for each amplicon. The plots contain the variant distribution per gene, distribution of confidence scores and variant types. The user can additionally download the visualization files for upload in the IGV and visually inspect the identified variants. The final report file contains high-quality variants ordered according to their confidence score.

### 3.2. VARIFI Validation and Evaluation

#### 3.2.1. Input File Examples

To test VARIFI, as well as optimize and evaluate its parameters, we used PGM output obtained by sequencing cancer biopsies. Each sample originated from a single biopsy from one patient. The patients were in an advanced stage of the disease and refractory to standard treatment regimens. We analyzed 170 patients. As for the bed file, we used Ion AmpliSeq Cancer Hotspot Panel v2^TM^ (Life Technologies) with 207 amplicons representing sequences of 50 genes. The median amplicon length was 109.0 bp. PGM produced on average 518,275.29 single end reads per sample, with an average read length of 108.72 bp (standard deviation (SD) = 7.07 bp).

#### 3.2.2. VARIFI Performance Evaluation

As an approximation of the time needed for the VARIFI pipeline to complete the analysis, we measured the VARIFI runtime for the smallest and the largest input (bam) file. The analysis of the file with the smallest input size (20 MB) lasted 4 h and 15 min, and the analysis of the largest file (265 MB) lasted 6 h and 42 min when we used 16 AMD Operon cores running at 2.8 GHz. The RAM high watermark was 11.76 Gb.

We compared the percentage of mapped reads of the three used aligners and found that bwa mapped on average 89.96% (SD = 3.40%), bowtie2 97.75% (SD = 1.65%) and NextGenMap 96.14% (SD = 3.62%) sequenced reads. VARIFI identified on average 17.66 (SD = 5.73) variants per patient sample.

To evaluate VARIFI performance and to compare it with Ion Reporter, we additionally sequenced 19 samples—13 samples using Ion AmpliSeq Cancer Hotspot Panel v2^TM^ and six samples using ColonLung Panel v2^TM^ (Life Technologies). We then separately analyzed sequenced samples by VARIFI and Ion Reporter. To calculate specificity and sensitivity, we picked the most commonly affected gene in these samples, *TP53*, and sequenced several exons of this gene by the Sanger method across all newly analyzed samples. Table S1 lists which exons were sequenced in each sample. Across 13 Cancer Hotspot Panel samples, 6300 Sanger-sequenced bases were considered for specificity and sensitivity calculations, whereas for the six samples analyzed with ColonLung Panel, 2886 bases were sequenced by the Sanger method. Table S1 shows that both Ion Reporter and VARIFI analyzed Cancer Hotspot Panel samples data with 100% specificity and 100% sensitivity. However, for ColonLung Panel samples VARIFI outperformed Ion Reporter—it analyzed the data with 100% specificity and 88.89% sensitivity, whereas Ion Reporter achieved 99.96% specificity and 87.50% sensitivity. Please not that we excluded from evaluation the variants found by VARIFI and Ion Reporter in Sanger-checked exons if their minor allelic frequency (MAF) was below 10%, because of limited sensitivity of the Sanger method.

To further explore the performance of VARIFI, we used the NA12878 sample, the most thoroughly analyzed sample available from the Genome in a Bottle (GIAB) [26] project. The aim of GIAB is the authoritative characterization of human genomes in order to support sequencing technology and genomic analysis improvements and benchmarking. For the VARIFI performance validation, we used reads produced by Ion Torrent technology (ftp://ftp-trace.ncbi.nih.gov/giab/ftp/data/NA12878/ion_exome/bb17523_PSP4_BC20.fastq) covering genomic regions listed in the bed file available at ftp://ftp-trace.ncbi.nih.gov/giab/ftp/data/NA12878/analysis/IonTorrent_TVC_06302015/AmpliseqExome.20141120_effective_regions.bed. To perform benchmarking, the authors of GIAB provide a vcf file containing a set of variants identified by several sequencing technologies and variant calling methods. We here refer to them as integrated calls, available at ftp://ftp-trace.ncbi.nih.gov/giab/ftp/data/NA12878/analysis/GIAB_integration/NIST_RTG_PlatGen_merged_highconfidence_v0.2_Allannotate.vcf.gz. The integrated calls are reported for the high confidence regions provided at: ftp://ftp-trace.ncbi.nih.gov/giab/ftp/data/NA12878/analysis/GIAB_integration/union13callableMQonlymerged_addcert_nouncert_excludesimplerep_excludesegdups_excludedecoy_excludeRepSeqSTRs_noCNVs_v2.19_2mindatasets_5minYesNoRatio_AddRTGPlatGenConf_filtNISTclustergt9_RemNISTfilt_RemPartComp_RemRep_RemPartComp_v0.2.bed.gz. This bed file excludes regions in which variant calling would be uncertain due to several reasons, such as low coverage, systematic sequencing errors, local alignment problems, mapping problems, etc. (a detailed description of the excluded regions is available at: ftp://ftp-trace.ncbi.nih.gov/giab/ftp/data/NA12878/analysis/GIAB_integration/README.GIAB.v0.2.txt).

We compared VARIFI results with integrated variant calls only in the high confidence regions for which we had Ion Torrent reads. VARIFI found a total of 80,099 variants, whereas the integration of different technologies found 30,316. The number of overlapping variants, obtained by the intersection of the VARIFI calls and the integrated call set, was 28,059, which means that VARIFI found 92.56% of integrated calls. The average confidence score of the overlapping variants was 5.79 (0.31% variants had a confidence score equal to 1, 0.52% variants had a confidence score equal to 2, 1.95% variants had a confidence score equal to 3, 4.61% variants had a confidence score equal to 4, 2.56% variants had a confidence score equal to 5, 90.04% variants had a confidence score equal to 6). VARIFI found an additional 52,040 variants that were not found in the integrated calls, and the variants had an average confidence score of 2.41 (30.60% variants had a confidence score equal to 1, 34.55% variants had a confidence score equal to 2, 15.94% variants had a confidence score equal to 3, 7.64% variants had a confidence score equal to 4, 4.57% variants had a confidence score equal to 5, 6.69% variants had a confidence score equal to 6). The number of variants found in the integrated calls, but not by VARIFI, was 2257. Presuming that the integrated calls present the truth, we calculated the sensitivity and specificity for the VARIFI calls with the confidence score above 4. For these variant calls, the sensitivity was 92.01% and the specificity was 99.98% (the number of TPs with a confidence score 5 and 6 was 25,983, the number of FPs with a confidence score of 5 and 6 was 5863, the number of FNs was 2257 and the number of TNs was 38,974,865, where TNs are bases in high confidence regions for which we had Ion Torrent reads, subtracted by a union of variants found by VARIFI and integrated calls). As stated above, the confidence score was used as a prioritizing criterion, and the variants with a higher confidence score (e.g., >4) have a higher chance of being true positives.

#### 3.2.3. Output File Examples

To check whether all amplicons were sequenced, VARIFI provides a coverage table with the number of reads mapped to each amplicon for the mappers we used (see an example of this file in Table S5).

**Figure 3:**
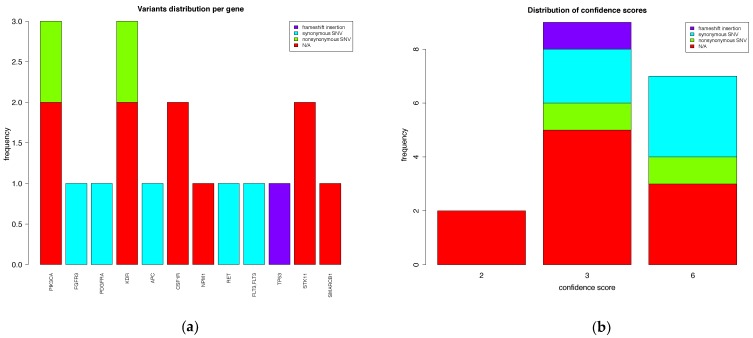
An example of VARIFI output plots. (**a**) Variant distribution on genes. In this example, the exonic function for most variants was not available (“N/A”) since the variants were found in the intronic and UTR3 regions, and the highest number of variants was found on *PIK3CA* and *KDR* genes, three variants on each gene. Five variants were synonymous single-nucleotide variants (SNVs), two variants were nonsynonymous SNVs and one variant was a frameshift insertion. (**b**) Distribution of variants based on their confidence score. Nine variants had a confidence score of 3, seven variants had a confidence score of 6 and two variants had a confidence score of 2.

Table S6 shows an example of a final report. Additionally, summary plots of the reported variants are provided. Figure 3a shows the distribution of variants per gene. We present the distributions of confidence scores of reported variants in Figure 3b with respect to their type.

## 4. Discussion

We introduced VARIFI to reliably find genetic variants in amplicon-sequenced (patient) samples. VARIFI is a web-based tool to carry out this analysis. We take into account biases connected to the Ion Torrent technology by providing a filter for incorrectly identified indels likely occurring in homopolymer regions. We minimized the number of falsely identified variants by using several filtering methods. All detected variants are automatically annotated and can be easily prioritized based on their potential effects and their confidence score. Recently [5], it was shown that an ensemble of different pipelines and methods gave a more accurate list of variants and thus outperformed the best individual pipeline. VARIFI applies the same strategy by combining several methods and assigning a confidence score to a called variant. We showed that the variants with a lower confidence score should not be ignored, but more confidence is given to those found by more aligner-variant caller combinations. We evaluated the performance of VARIFI by Sanger sequencing on *TP53* exons in 13 samples analyzed with Cancer Hotspot Panel, where VARIFI performed with 100% specificity and 100% sensitivity. Moreover, in six samples with ColonLung Panel, VARIFI analyzed data with 100% specificity and 88.89% sensitivity. We believe that the low sensitivity is probably due to the small number of samples we analyzed with Sanger sequencing. In addition, we compared VARIFI calls with the integrated calls available for the sample NA12878. VARIFI found 92.56% integrated calls, with a sensitivity of 92.01% and a specificity of 99.98% for the VARIFI calls with a confidence score >4.

The time required to obtain results ranged from four to seven hours, which makes VARIFI suitable for clinical usage, where fast and trustworthy variant identification can support diagnostics and therapy decision. VARIFI is meant to provide a structured overview of variants found in the regions of interest and to be used only as a support to clinical decisions, to strike gene alterations that could be potentially pathogenic. It is not meant to be a decision-making tool in treating patients, but to assist practitioners in disease diagnostics. The use of the VARIFI server should comply with local and international health data privacy policies and regulations.

As shown in a recent publication [27], it is still necessary to check the potentially disease-causing variants with Sanger sequencing, and VARIFI was created to help with the selection of variants which should be inspected with other clinically accepted decision-making methods. The current limitations of the VARIFI pipeline, which we plan to address in the future VARIFI releases, include the relatively older versions of the utilized alignment and variant calling tools and the current file size input of 400 MB. Additionally, our future work will include the optimization of the runtime and performance of VARIFI as well as its adaptation for outputs created by other sequencing platforms.

## Supplementary Materials

The following are available online at https://www.mdpi.com/2075-4426/9/1/10/s1, Table S1: Comparisons of variant calling results using VARIFI and Ion Reporter, Table S2: FP variants called in homopolymer regions—sample S_1_, Table S3: FP variants called in homopolymer regions—sample S_2_, Table S4: Validation of variants found with different confidence scores, Table S5: Amplicon coverage, Table S6: Example of a final report, File S1: An example of *var* and *frmode* calculation.

## References

[1] Meldrum C., Doyle M.A., Tothill R.W. (2011). Next-generation sequencing for cancer diagnostics: A practical perspective. Clin. Biochem. Rev. Aust. Assoc. Clin. Biochem..

[2] Rehm H.L. (2013). Disease-targeted sequencing: A cornerstone in the clinic. Nat. Rev. Genet..

[3] Mendez P., Dang J., Kim J.W., Lee S., Yoon J.H., Kim T., Sailey C.J., Jablons D.M., Kim I.J. (2016). Comprehensive evaluation and validation of targeted next-generation sequencing performance in two clinical laboratories. Int. J. Oncol..

[4] Gleeson F.C., Voss J.S., Kipp B.R., Kerr S.E., Van Arnam J.S., Mills J.R., Marcou C.A., Schneider A.R., Tu Z.J., Henry M.R. (2017). Assessment of pancreatic neuroendocrine tumor cytologic genotype diversity to guide personalized medicine using a custom gastroenteropancreatic next-generation sequencing panel. Oncotarget.

[5] Ewing A.D., Houlahan K.E., Hu Y., Ellrott K., Caloian C., Yamaguchi T.N., Bare J.C., P’ng C., Waggott D., Sabelnykova V.Y. (2015). Combining tumor genome simulation with crowdsourcing to benchmark somatic single-nucleotide-variant detection. Nat. Methods.

[6] Alioto T.S., Buchhalter I., Derdak S., Hutter B., Eldridge M.D., Hovig E., Heisler L.E., Beck T.A., Simpson J.T., Tonon L. (2015). A comprehensive assessment of somatic mutation detection in cancer using whole-genome sequencing. Nat. Commun..

[7] Li H., Durbin R. (2009). Fast and accurate short read alignment with burrows-wheeler transform. Bioinformatics.

[8] Langmead B., Salzberg S.L. (2012). Fast gapped-read alignment with bowtie 2. Nat. Methods.

[9] Sedlazeck F.J., Rescheneder P., von Haeseler A. (2013). Nextgenmap: Fast and accurate read mapping in highly polymorphic genomes. Bioinformatics.

[10] Li H., Handsaker B., Wysoker A., Fennell T., Ruan J., Homer N., Marth G., Abecasis G., Durbin R., Genome Project Data Processing Subgroup (2009). The sequence alignment/map format and samtools. Bioinformatics.

[11] McKenna A., Hanna M., Banks E., Sivachenko A., Cibulskis K., Kernytsky A., Garimella K., Altshuler D., Gabriel S., Daly M. (2010). The genome analysis toolkit: A mapreduce framework for analyzing next-generation DNA sequencing data. Genome Res..

[12] Sherry S.T., Ward M.H., Kholodov M., Baker J., Phan L., Smigielski E.M., Sirotkin K. (2001). dbSNP: The NCBI database of genetic variation. Nucleic Acids Res..

[13] Wang K., Li M., Hakonarson H. (2010). Annovar: Functional annotation of genetic variants from high-throughput sequencing data. Nucleic Acids Res..

[14] Genomes Project C., Abecasis G.R., Auton A., Brooks L.D., DePristo M.A., Durbin R.M., Handsaker R.E., Kang H.M., Marth G.T., McVean G.A. (2012). An integrated map of genetic variation from 1,092 human genomes. Nature.

[15] Adzhubei I.A., Schmidt S., Peshkin L., Ramensky V.E., Gerasimova A., Bork P., Kondrashov A.S., Sunyaev S.R. (2010). A method and server for predicting damaging missense mutations. Nat. Methods.

[16] Schwarz J.M., Rodelsperger C., Schuelke M., Seelow D. (2010). Mutationtaster evaluates disease-causing potential of sequence alterations. Nat. Methods.

[17] Siepel A., Bejerano G., Pedersen J.S., Hinrichs A.S., Hou M., Rosenbloom K., Clawson H., Spieth J., Hillier L.W., Richards S. (2005). Evolutionarily conserved elements in vertebrate, insect, worm, and yeast genomes. Genome Res..

[18] Davydov E.V., Goode D.L., Sirota M., Cooper G.M., Sidow A., Batzoglou S. (2010). Identifying a high fraction of the human genome to be under selective constraint using GERP++. PLoS Comput. Biol..

[19] Kumar P., Henikoff S., Ng P.C. (2009). Predicting the effects of coding non-synonymous variants on protein function using the sift algorithm. Nat. Protoc..

[20] Forbes S.A., Beare D., Gunasekaran P., Leung K., Bindal N., Boutselakis H., Ding M., Bamford S., Cole C., Ward S. (2015). Cosmic: Exploring the world’s knowledge of somatic mutations in human cancer. Nucleic Acids Res..

[21] Thorvaldsdottir H., Robinson J.T., Mesirov J.P. (2013). Integrative Genomics Viewer (IGV): High-performance genomics data visualization and exploration. Brief. Bioinform..

[22] Liu L., Li Y., Li S., Hu N., He Y., Pong R., Lin D., Lu L., Law M. (2012). Comparison of next-generation sequencing systems. J. Biomed. Biotechnol..

[23] Bragg L.M., Stone G., Butler M.K., Hugenholtz P., Tyson G.W. (2013). Shining a light on dark sequencing: Characterising errors in ion torrent pgm data. PLoS Comput. Biol..

[24] Yeo Z.X., Chan M., Yap Y.S., Ang P., Rozen S., Lee A.S. (2012). Improving indel detection specificity of the ion Torrent PGM benchtop sequencer. PLoS ONE.

[25] Yeo Z.X., Wong J.C., Rozen S.G., Lee A.S. (2014). Evaluation and optimisation of indel detection workflows for ion torrent sequencing of the *BRCA1* and *BRCA2* genes. BMC Genom..

[26] Zook J.M., Chapman B., Wang J., Mittelman D., Hofmann O., Hide W., Salit M. (2014). Integrating human sequence data sets provides a resource of benchmark snp and indel genotype calls. Nat. Biotechnol..

[27] Mu W., Lu H.M., Chen J., Li S., Elliott A.M. (2016). Sanger confirmation is required to achieve optimal sensitivity and specificity in next-generation sequencing panel testing. J. Mol. Diagn. JMD.

