# Supplementary material for: VARIFI—Web-Based Automatic Variant Identification, Filtering and Annotation of Amplicon Sequencing Data
Source: J Pers Med. 2019 Feb 1;9(1):10. doi: 10.3390/jpm9010010 (PMC6463100; doi:10.3390/jpm9010010)
Supplement: Supplementary file 1 [file jpm-09-00010-s001.zip › File_S1.pdf]

# An example of calculating *var* and *frmode*

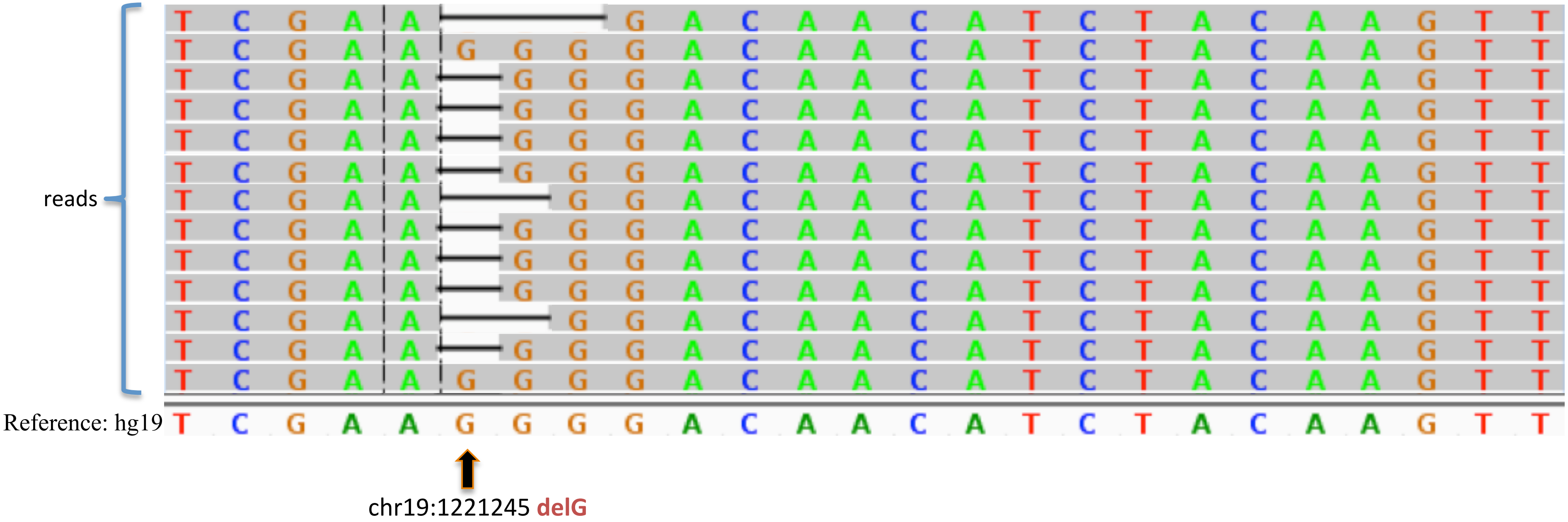


N = number of reads containing indel (deletion of G in this example) at the indel position (e.g. chr19:1221245)= 11
X= vector of size N containing indel (deletion) lengths = [1,2,1,1,1,2,1,1,1,1,3]
μ = mean of indel lengths = (1+2+1+1+1+2+1+1+1+1+3)/11=1.36
*var* = variance of indel length (formula at the bottom right) = 0.41
Mode of distribution of indel lengths = the most common indel(deletion) length at the indel position =1
*frmode* of indel lengths = frequency of the most common indel length at that position: in 8 out of 11 reads the most common indel length is 1, therefore frmode = 8/11 = 0.73.

$$\sigma^2 = \frac{\sum (X-\mu)^2}{N}$$
